# A novel one arm motorized walker for hemiplegic stroke survivors: a feasibility study

**DOI:** 10.1186/s12938-018-0446-z

**Published:** 2018-01-30

**Authors:** Ki-Hun Cho, SeungHyeon Pyo, Gi-Su Shin, Sung-Duk Hong, Se-Han Lee, DongGeon Lee, SunHae Song, GyuChang Lee

**Affiliations:** 1 Department of Physical Therapy, Korea National University of Transportation, Chungju, 27469 Republic of Korea; 2 Department of Physical Therapy, Graduate School of Kyungnam University, Changwon, 51767 Republic of Korea; 3 Anytoy Co., Ltd., Changwon, 51233 Republic of Korea; 4 Department of Mechanical Engineering, Kyungnam University, Changwon, 51767 Republic of Korea; 5 Department of Physical Therapy, Kyungnam University, 7 Kyungnamdaehak-ro, Masanhappo-gu, Changwon, Gyeongsangnam-do 51767 Republic of Korea; 6 Department of Rehabilitative & Assistive Technology, National Rehabilitation Research Institute, National Rehabilitation Center, Seoul, 01022 Republic of Korea

**Keywords:** One-arm motorized walker, Hemiplegic stroke survivor, Gait

## Abstract

**Background:**

A hemiplegic stroke survivor with a moderate to severe gait disturbance may have difficulty walking using a one-arm walker. This study aimed to test the safety and feasibility of a prototype one-arm motorized walker that uses a power-driven device to provide gait assistance to hemiplegic stroke survivors with moderate to severe gait disturbances.

**Methods:**

A one-arm motorized walker with a power-driven device was developed and tested with respect to 10 distinct variables, including weight, degrees of freedom, handle, handle substitution function, two-sided use function, variable handle height, redirecting function, electric moving parts through the handle control, brake function using the handle control, folding chairs, and design stability. Its safety and feasibility were tested in 19 hemiplegic stroke individuals using the Likert scale and a simple interview.

**Results:**

The walker consists of a frame platform including a handle, electric motor for driving, one wheel for driving, two wheels for turning, unlocking sensor, driving button, and turning buttons. The walker is programmed so that a touch sensor in the handle can unlock the locking system. Furthermore, it is programmed so that a user can propel it by pushing the handle downward or pressing a button and can control directions for turning right or left by pressing buttons. Safety and performance testing was achieved for 10 separate variables, and a Likert scale score of 3.5 of 5 was recorded.

**Conclusion:**

This walker’s novel design was developed for hemiplegic stroke survivors with moderate to severe gait disturbances. Our findings indicate that the walker is both safe and feasible for providing walking assistance to hemiplegic stroke survivors and establish the potential advantages of the one-arm motorized walker.

## Background

Stroke is a leading cause of disability and mortality in the adult population worldwide [1]. As many as 88% of stroke patients experience hemiparesis [2, 3]. Hemiparesis induces an asymmetric gait that negatively influences one’s independent activities of daily living (ADL) by increasing energy expenditures and reducing activity levels [4–6].

The general characteristics of hemiparetic gait include reduced propulsion of the more affected side, stance phase duration, step length of the less affected side, and gait speed [7]. More than 80% of stroke survivors face difficulty ambulating in the community due to gait deficits [8]. Thus, addressing gait disorders is essential to survivor rehabilitation and improving quality of life [9, 10]. The goals of stroke rehabilitation are enhancing walking ability and boosting ADL participation [11, 12]. However, only 64% of stroke survivors can walk independently after undergoing rehabilitation treatment [13].

To compensate for abnormal gait patterns, most stroke survivors depend on walking aids [14]. A reported 76% of stroke survivors have used at least one walking aid after post-stroke rehabilitation [15, 16]. Walking aids not only increase postural stability and muscle action in survivors with neurological disorders and decrease weight loads on the lower extremity of the more affected side [17, 18], they may also decrease the chance of falling [19]. Walking aids include walkers, hemi-walkers, one-point sticks, three- or four-point (quad) canes, crutches, and ankle–foot orthoses [20]. One study reported that > 67% of stroke survivors use a cane as a walking aid [21].

A previous study comparing the efficiency of single-point canes, quad canes, and hemi-walkers found that oxygen expenditure, gait endurance, and gait velocity are higher with single-point cane use than with quad or hemi-walker cane use [14]. These results suggest that the use of a single-point cane is more effective as it decreases the oxygen requirement more quickly and is easy to handle and maneuver than a quad cane or a hemi-walker.

Although canes and walkers might be helpful for stroke survivors with stable gait patterns in the chronic stage [22, 23], their use is difficult or improper for hemiparetic survivors who have difficulty concomitantly controlling their upper and lower limbs or have different muscle tones of the upper and lower limbs [24]. Thus, a user’s gait can be effectively aided by the addition of a motorized system to the walker to adjust driving and steering.

Thus, this study sought to develop and test the feasibility of a one-arm motorized walker that uses a power-driven device to provide gait assistance and train hemiparetic stroke survivors by providing the ability to overcome the limitations in existing walking aids.

## Methods

### Development of the one-arm motorized walker

#### Mechanical system

**Fig. 1:**
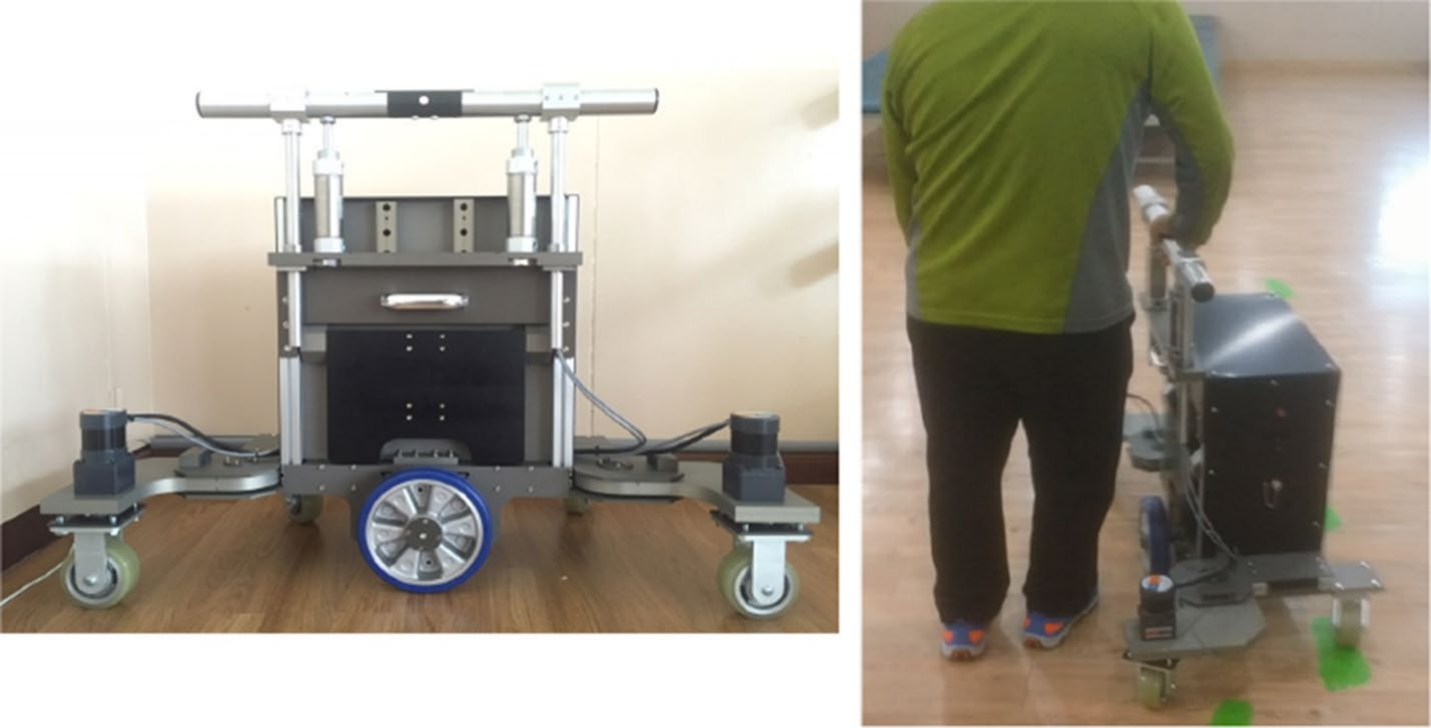
A study participant with hemiplegic stroke walked with one arm motorized walker

**Fig. 2:**
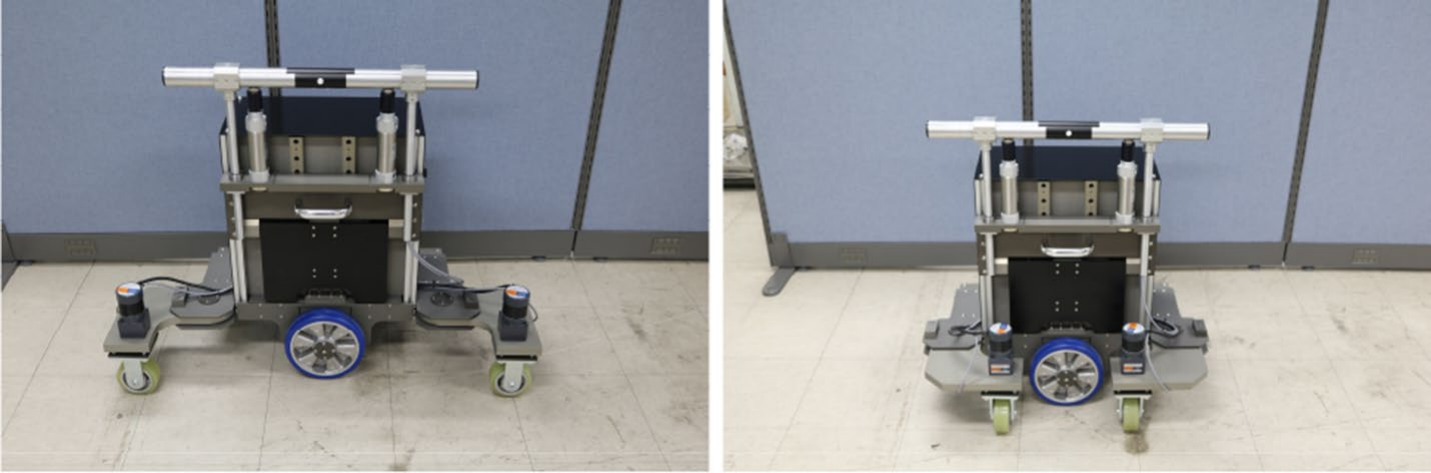
Folding the steering wheel

**Fig. 3:**
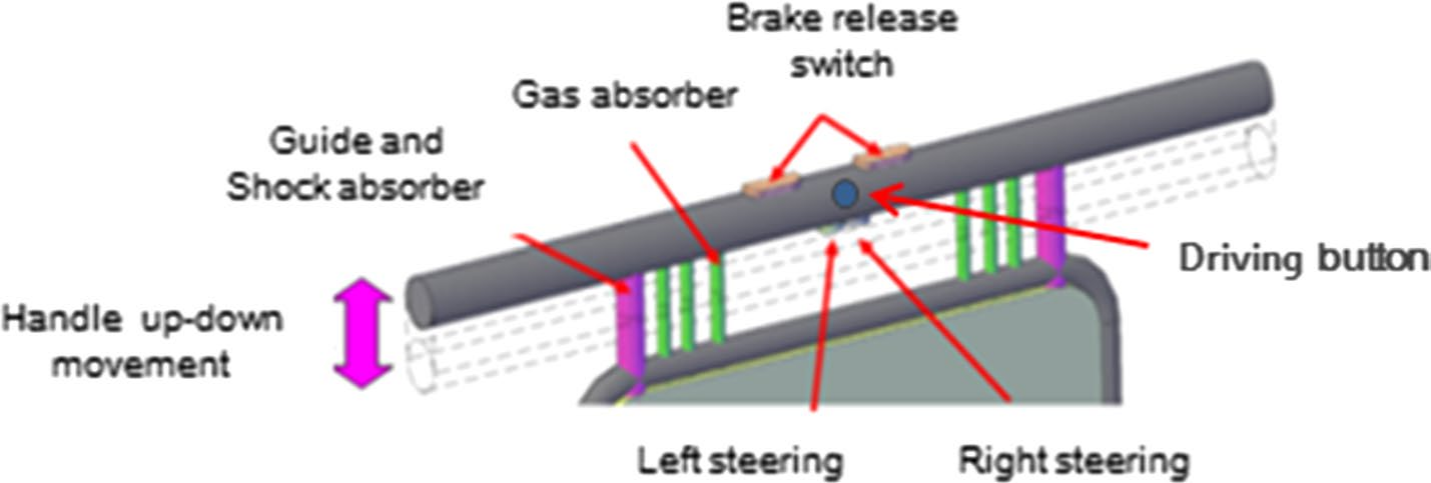
The handle part of one arm motorized walker

**Fig. 4:**
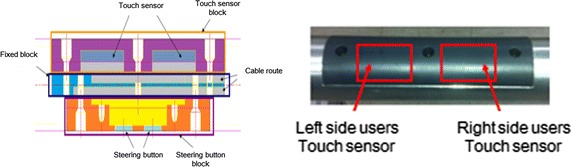
A touch-sensor installed at the inner part of handle and equipped at the top of handle has two touch-sensors, each for left-handed and right-handed

**Fig. 5:**
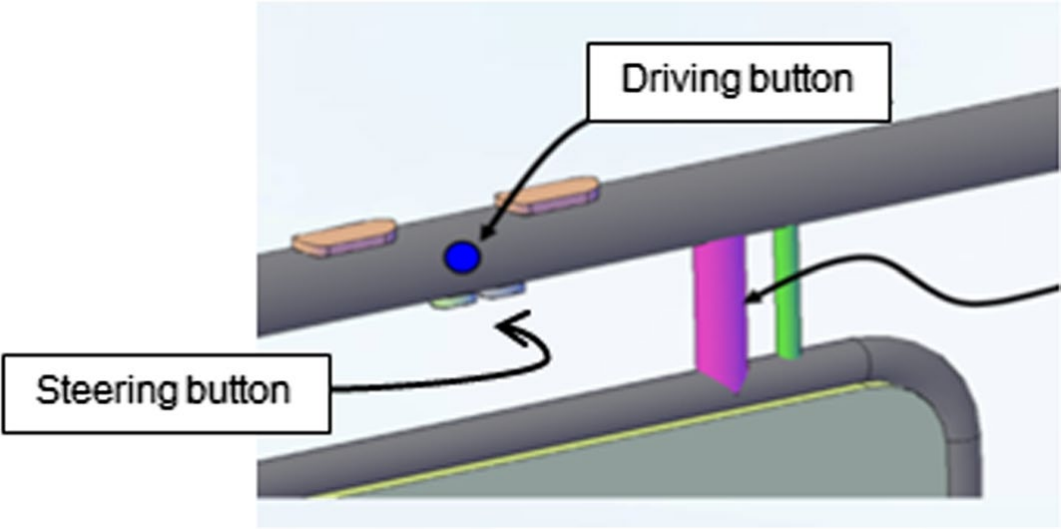
Push buttons located at the side of the handle for moving forward and located at the bottom of the handle for steering the wheel left or right

The one-arm motorized walker was developed for rehabilitation training and gait assistance for hemiplegic stroke survivors. The walker consists of a frame platform, handle, electric motor for driving, one wheel for driving, two wheels for turning, an unlocking sensor, a button for driving, and buttons for turning (Fig. 1). Figures 2, 3, 4, 5 show the detailed mechanical system of the one-arm motorized walker.

A driving wheel (200-W motor) was installed at the bottom center of the body to provide support, while the stability of the body was secured by positioning a steering wheel in front of or behind the user and installing a caster outside the body. To enhance mobility, the steering wheel folds for loading into a vehicle (Fig. 2).

The handle was designed to be 40 mm in diameter with an adjustable height for ease of handling. The walker was manufactured with two-way measures to enable touch-tone driving and steering using a button at the handle (Fig. 3).

For operational measurements, a touch sensor was installed at the inner part of the handle to activate the system. The top of the handle has two touch sensors, one each for left- and right-handed users, and the system stops operating for safety reasons when released (Fig. 4).

In addition, a push button located at the side of the handle controls forward motion, while another located at the bottom of the handle steers the wheels left or right (Fig. 5).

To minimize the user’s interaction space, the platform structure was simplified to secure a competitive price based on the simplified internal/external components and the user-friendly design. The lightweight frame (< 45 kg) provides a solid structure that prevents falling. The wheels were also equipped for straight or side-to-side driving and to negotiate obstacles > 10 mm in height. A folding seat was also added for user convenience.

#### Controls and algorithms

Several control algorithms are used by the one-arm motorized walker, which is programmed so that a touch sensor in the handle portion can unlock the system. Therefore, the system is unlocked and allowed to work only when a user touches the touch sensor in the handle portion since the walker is originally locked to prevent unintentional working. It is also programmed so that a user can propel the walker by pushing the handle portion downward or pressing a button. In other words, the driving wheels in the middle of the bottom of the walker start moving, thereby creating a forward-moving walk when a user pushes the handle portion downward or presses the start button on the side of the handle portion. In addition, two steering wheels located at the front and the back of the walker start turning right or left when the steering button on the bottom of the handle portion in pressed, which allows a user to turn the walker right or left. Directional control is also initiated via a button on the handle. Furthermore, the walker ensures that people who have difficulties with gait can use it stably by preventing the simultaneous occurrence of driving and steering, which helps prevent unintended directional changes while driving.

An industrial programmable logic controller (PLC) was used to provide stability to the control device. As the working voltage of the PLC is DC 24 V, a lead-acid battery of that power was used for mobility. The application of an extended battery pack in a slotted-type case secured the operating range of 1–4 h and provides ease of detachment and maintenance. As the fully charged voltage of the battery was 28–30 V, it was built to provide a stable supply of 24 V using a DC-to-DC converter to protect the PLC and motor drive. A digital automatic charger that outputs 27.4–29.4 V was used to charge an empty battery, and the standardized interfaces for external charge were installed.

In addition, as the completely discharged battery cannot be used and must be discarded, the controller equipped with a charge–discharge protection circuit was used to prevent overdischarge. It was designed to move forward or left and right by using steering motors and driving motors from the PLC input to notify the states of the sensors and switches attached to the handle.

#### Performance features of the one-arm motorized walker

Performance testing of the walker was conducted with respect to ten factors: weight, degree of freedom, handle separation or substitution function, two-sided use function, handle height adjustment, redirecting function, electric moving parts through the handle control, brake function using the handle control, folding chairs, and design stability.

The development goal of a unit weight < 45 kg was met with an overall final weight of 44 kg. The walker has two degrees of freedom with respect to driving and steering. In addition, in one of the development goals, we sought to include separation or substitution functions as well as a two-sided use function. The purpose of the separation or substitution function is to separate and replace the handle when storing the walker in a narrow space or the handle is out of order rather than replacing the entire walker body. The handle was designed with a range of 790–940 mm (interval value of 865 ± 75 mm between the 5th and 95th percentiles) corresponding to the average Korean individual’s fist height of 744.13 mm. In addition, a redirecting function was included and developed in accordance with the development goal. A speed control function of the electric moving part through the handle control was developed to have a speed of 0.4 m/s, the speed required for independent indoor walking, and 0.4–0.8 m/s, the speed required for pedestrians who walk in a limited local area [25]. This function adopts a modulating control method. In this method, speed slowly increases from 0 to 2.5 km/h when the user presses a button but slowly decreases when the hand is removed from the button, which met the development goal of 0–2.5 km/h. The brake function, which is operated by manipulation of the handle portion, was developed to comply with 7°, the brake operation force required by electric wheelchair items according to Korean standards. The floor height of the folding seat was 415 mm, which satisfied the design criteria of > 350 mm. The design of a tool to prevent overturning during a stationary state was included in the development goal. Thus, it was concluded that the one-arm motorized walker met the ten factors of the development goal.

### Safety and feasibility study

#### Methods

The safety and feasibility test of a one-arm motorized walker was conducted three times with 19 hemiplegic stroke survivors hospitalized at H Rehabilitation Hospital in Changwon. The first safety and feasibility test was conducted at the development stage, while the second and third safety and feasibility tests were conducted after the first product improvement and the final development, respectively. The participants were recruited by a public notice posted inside the hospital. The final participants were chosen in accordance with the following selection criteria: hemiplegic stroke survivor, ability to walk for > 1 m with or without assistance, and ability to follow the directions provided by the researcher.

Among the 10 participants who wanted to participate in the first safety and feasibility test, six eventually participated. Two participants were excluded from the test because they could not walk > 1 m, whereas another two were excluded because they could not follow the researcher’s instructions. Participants used the one-arm motorized walker in an indoor environment. They walked straight to a triangular obstacle located 3 m from the starting line, turned around the obstacle, and walked back to the starting line with the assistance of the one-arm motorized walker. To avoid having the walker be interrupted by an obstacle during the motion of turning around, left hemiplegic stroke survivors were instructed to turn to the left and right hemiplegic stroke survivors were instructed to turn to the right. The participants were asked about their satisfaction with using the one-arm motorized walker. Satisfaction was assessed from 1 to 5 points on a Likert scale. In addition, a brief interview was conducted to investigate the participants’ acceptability of and discomfort when using the developed walker.

For the second safety and feasibility test, 10 of 11 survivors participated. One survivor was removed from the study due to an inability to comply with the instructions due to a language barrier. The second test was conducted in the same way as the first test after the one-arm motorized walker was improved according to results of the first safety and feasibility test.

For the third safety and feasibility test, among the five hemiplegic stroke survivors, two were excluded from the study because they were unable to walk for > 1 m. Thus, three hemiplegic stroke survivors performed the safety and feasibility test. The third test was conducted in the same way as the second test after the one-arm motorized walker was improved in accordance with the results of the second safety and feasibility test.

## Results

Participant 1, who participated in the first safety and feasibility test, responded that the walker seemed to have difficulty driving over small obstacles and advancing on low inclined surfaces due to the small wheel size. This participant reported a 3 on the Likert scale. Participant 2 answered that the walker should be modified for easy storage and movement by a size reduction or folding ability and also reported a 3 on the Likert scale. Participants 3 and 4, whose Likert scale scores were 3, complained of inconvenience caused by storage difficulties due to the walker’s size and tripping over it due to the narrow space between the front and rear wheels. Participant 5 responded that the walker was relatively stable compared with existing walking aids. However, this participant experienced anxiety due to unnatural redirection. This patient’s Likert scale score was 4.

For the second safety and feasibility test, participants 1 and 2, whose Likert scale scores were 3, complained of fast wheel speed while turning, although they said that the improvements made after the first test improved driving stability. Participants 3 and 4 also experienced turning difficulties due to the faster wheel speed and reported scores of 2 and 3 on the Likert scale, respectively. Conversely, participants 5 and 6 cited storage difficulties due to walker size but admitted that its weight provided stability. Their Likert scale scores were 3. Participants 7 and 8 responded that they wanted quick commercialization of the walker because they were highly satisfied with its stable driving ability. Their Likert scale scores were 4. Participant 9, whose Likert scale score was 2, cited inconvenience caused by the walker’s large size and heavy weight and suggested that LED lights would be useful for mobility in a dark indoor area. Participant 10 responded that controlling the button might be burdensome for people with cognitive problems and cited a Likert scale score of 3.

The Likert scale score of participant 1, who participated in the third safety and feasibility test, was 3. He assigned satisfactory scores to the driving and steering controls; however, he had issues with tripping on the more affected side of his body because of wheel instability when switching directions. The Likert scale score of participant 2 was also 3. Although he cited slight difficulty with driving the touch-tone and steering control, he stated that it was stable enough to support his weight and reduce his fear of falling. The Likert scale score of participant 3 was 5, and he stated feeling discomfort with the location of the steering and button controls.

During the safety and feasibility testing process, the satisfaction score of the first test was 3.33 of 5, while the satisfactory scores of the second and the third tests were 3 and 3.5 of 5, respectively. The participants were generally satisfied with the walker’s stable driving ability provided by its weight but complained of difficulty storing the walker because of its large size and the inconvenience caused by contact between the user’s foot and the rear wheel during walker-assisted gait.

## Discussion

A reduced ability to control movements is a major cause of falls in stroke survivors [26], which may result in secondary impairment [27]. It is a critical factor for stroke survivors because it limits their ability to live independently [28]. Because the loss of mobility influences a stroke survivor’s ability to perform ADL and makes them dependent on others, it is critical to improve their walking ability during rehabilitation [29, 30] and provide a higher class of ambulation to enhance their quality of life [31].

Nevertheless, only 64% of survivors recovered their independent gait function after rehabilitation, 14% could walk with assistance, 22% showed no recovery in their gait function, and > 50% of the stroke survivors underwent continuous rehabilitation to regain functional ambulation [13]. Most stroke survivors reportedly used more than one walking aid after stroke [15, 16] and relied on walking aids, including walkers and canes, to increase their stability and maintain walking ability [14].

Existing walking aids, including canes and walkers, can be used only if voluntary movement is possible since they do not feature power units. In the case of walkers with wheels that lack a braking system, the user must lift the entire device when walking. In addition, there are restrictions for hemiplegic stroke survivors using their existing walking aids due to friction. Thus, it is important to develop devices that can assist and support elderly and disabled individuals with reduced walking abilities [29].

Maria et al. studied the robotic technology related to mobility assistive devices for participants with mobility disabilities [29]. The smart cane developed at Massachusetts Institute of Technology collects a user’s velocity and direction information by equipping a pair of three-dimensional force sensors and allowing participants to avoid danger by detecting obstructions. The guide cane is developed for visually impaired users to detect and avoid hazards using global positioning system and ultrasound sensors. In addition, the I-Walker, developed by Annicchiarico et al. is a robotic collator equipped with integrated sensors and actuators [32]. The characteristics of this walker include an ability to analyze real-time information related to forces from the handlebars and the floor and the relative position of the wheels on speed, a motor that applies two strategies for moving and braking, and a navigation functionality that is composed of cognitive modules that give commands to assist indoor users. The equipped sensors increase or decrease the walker’s velocity by recognizing the slope of the path. When a user’s fall risk increases, the navigator identifies a different path. Additionally, when movement is stopped due to obstructions, the device guides the user to a new path or requests assistance [29, 32].

There are several other types of assistive walking devices, including the personal adaptive mobility aid walker, which provides assistant-enabled direction control of the front wheels and a fully manual mode; the MARC smart walker, which is equipped with laser/infrared sensors on the forward bumper, which recognize steps or obstructions in the driving paths; and the personal aid for mobility and health monitoring system smart walker, which monitors the health of users who are in an elderly care facility. Assistive devices including smart canes and walkers are designed to relieve pathological gait, enhance balance, and decrease the load applied to the lower limbs by supporting the upper limbs as well as prevent early wheelchair use by preserving a user’s remaining locomotive capability.

Here we developed a one-arm motorized walker that features a transmission gear to assist with gait training of hemiplegic stroke survivors, who have reduced mobility, and support their ability to perform ADL. We then evaluated its efficiency and feasibility. In terms of driving and steering of the motorized device using buttons, the one-arm motorized walker enabled users to drive and steer in the direction of their choice using the fingers only on the condition that the hand is not separated from the handle without the discomfort of moving the assistive device by lifting it up. This design increased user convenience by controlling the user’s button operating time. The addition of a clip type hand-operated brake provided safety, while the three-point support design provided stability, thereby preventing falls. In addition, the developed one-arm motorized walker design was studied to satisfy the development goals based on the performance test consisting of 10 items. The safety and feasibility test was conducted three times to investigate the satisfaction of hemiplegic stroke survivors, and the results were expressed as 5-point Likert scale scores of 3.33 in the first test, 3 in the second test, and 3.5 in the third test (mean 3.28). In addition, the participants were generally satisfied with the stable driving conferred by the walker due to its weight, but they complained of difficulty storing it due to its large size and the inconvenience caused by contact between the user’s foot and the rear wheel while walking. We concluded that all users were satisfied with the walker’s stability and mobility; however, they had difficulty with tripping on the more affected side because of the rear wheel and the touch-tone control.

In reality, during rehabilitation programs designed for gait and balance recovery, many people use both traditional assistive devices and rehabilitation devices. Compared with traditional assistive devices, which require great force to use, the one-arm motorized walker developed in this study makes it possible to use only the motorized system, touch sensor, and button control system. This is can be an excellent gait training device for hemiplegic stroke survivors with reduced walking ability because it involves a lighter workload than do traditional assistive devices. Therefore, it will not only help the disabled individuals and stroke survivors but also enhance the quality of life of their family members by enabling users’ independent mobility. It is also expected to effectively reduce social burdens and medical costs.

The results of this study demonstrated that the walker is safe and feasible. Furthermore, the study highlighted the potential advantages of the one-arm motorized walker, the first of its kind, for providing walking assistance or rehabilitation training to hemiplegic stroke survivors. At the time this study was conducted, one-arm motorized walkers were not commercially available.

However, according to the participants’ opinions, compared with traditional assistive devices, the developed one-arm motorized walker is limited by its larger size, which makes it difficult to move and carry. Thus, it will be necessary to consider improvements that later increase weight but reduce volume and prevent possible rollovers. In addition, the satisfaction rate implies that feet tripping over the rear wheel and associated psychological anxiety are additional barriers to the use of this device. However, the participants may have felt discomfort using the controls because they were stroke survivors with neurological disorders. Because of such disadvantages, the ability to adjust the front and rear wheels is required. The design of a manual on/off operation of the one-arm motorized walker should be studied in the future. In addition, future studies should investigate various types of neurological disorders and the walker’s usability should be confirmed in high-quality clinical gait analysis.

## Conclusion

This one-arm motorized walker is a novel design developed for the rehabilitation or assistance of gait of hemiplegic stroke survivors with moderate to severe gait disturbances. This study demonstrated that the walker is safe and feasible and indicated several potential advantages. At the time of this study, one-arm motorized walkers were not commercially available. This walker is the first of its kind to provide walking assistance or rehabilitation training for hemiplegic stroke survivors. Thus, further studies, such as a gait analysis and a high-quality randomized control trial, are needed to demonstrate its usability and effectiveness.
